# Classic case report: concomitant pulsed field ablation, cavotricuspid isthmus ablation and percutaneous left atrial appendage occlusion in a patient with prior intracranial haemorrhage

**DOI:** 10.1093/ehjcr/ytag742

**Published:** 2026-10-07

**Authors:** Rahul G Muthalaly, Sara El-Saadany, Richard Ang, Mehul B Dhinoja, Mark J Earley

**Affiliations:** Barts Heart Centre, St Bartholomew’s Hospital, W Smithfield, London EC1A 7BE, United Kingdom; Barts Heart Centre, St Bartholomew’s Hospital, W Smithfield, London EC1A 7BE, United Kingdom; Barts Heart Centre, St Bartholomew’s Hospital, W Smithfield, London EC1A 7BE, United Kingdom; Barts Heart Centre, St Bartholomew’s Hospital, W Smithfield, London EC1A 7BE, United Kingdom; Barts Heart Centre, St Bartholomew’s Hospital, W Smithfield, London EC1A 7BE, United Kingdom

**Keywords:** Atrial fibrillation, Left atrial appendage occlusion, Pulsed field ablation, Ischaemic stroke, Case report

## Abstract

**Background:**

Concomitant atrial fibrillation (AF) ablation and percutaneous left atrial appendage occlusion (pLAAO) offers single-procedure rhythm control and stroke prevention in patients unable to tolerate long-term oral anticoagulation. Patients with prior intracranial haemorrhage (ICH) from suspected cerebral amyloid angiopathy (CAA) have among the strongest contraindications to anticoagulation yet are absent from randomized trials of concomitant procedures, and combining pulsed field ablation (PFA) with pLAAO introduces energy-specific considerations.

**Case Summary:**

A 66-year-old woman with persistent AF, cavotricuspid isthmus–dependent atrial flutter and a CHA_2_DS_2_-VA score of 3 had been off anticoagulation for 2 years after an ICH. Brain magnetic resonance imaging showed microhaemorrhages and siderosis suggestive of CAA. Through a single transseptal puncture, four-vein PFA was performed first to avoid electrical interaction with the nitinol pLAAO frame, followed by WATCHMAN FLX deployment under transoesophageal echocardiographic and computed tomography (CT)–fluoroscopy guidance and radiofrequency cavotricuspid isthmus ablation. She received edoxaban without aspirin for three months. Cardiac CT then confirmed device seal without device-related thrombus, and all antithrombotic therapy was withdrawn.

**Discussion:**

Randomized trials show that anticoagulation after ICH prevents ischaemic stroke at the cost of a marked increase in recurrent haemorrhage, and lobar or CAA-related haemorrhage carries the least favourable balance. Concomitant ablation and pLAAO delivers rhythm control and stroke prevention with one transseptal access and one time-limited exposure to anticoagulation. Combining PFA with pLAAO requires sizing the occluder before ablation, because PFA induces acute pulmonary ridge oedema, and completing pulmonary vein isolation before deployment to avoid arcing against the nitinol frame.

Learning points

- In patients with atrial fibrillation and cerebral amyloid angiopathy, oral anticoagulation is generally contraindicated, and even antiplatelet therapy carries haemorrhagic risk.
- Antithrombotic therapy after appendage occlusion in this group must be individualized—a short course of a direct oral anticoagulant, followed by complete withdrawal, is a pragmatic approach.

## Introduction

Concomitant atrial fibrillation (AF) ablation and percutaneous left atrial appendage occlusion (pLAAO) offer rhythm control and stroke prevention in a single procedure. We report concomitant pulsed field ablation (PFA), cavotricuspid isthmus ablation, and pLAAO in a patient in whom a prior intracranial haemorrhage (ICH) with suspected cerebral amyloid angiopathy (CAA) precluded long-term oral anticoagulation (OAC).

## Summary figure

**Figure:**
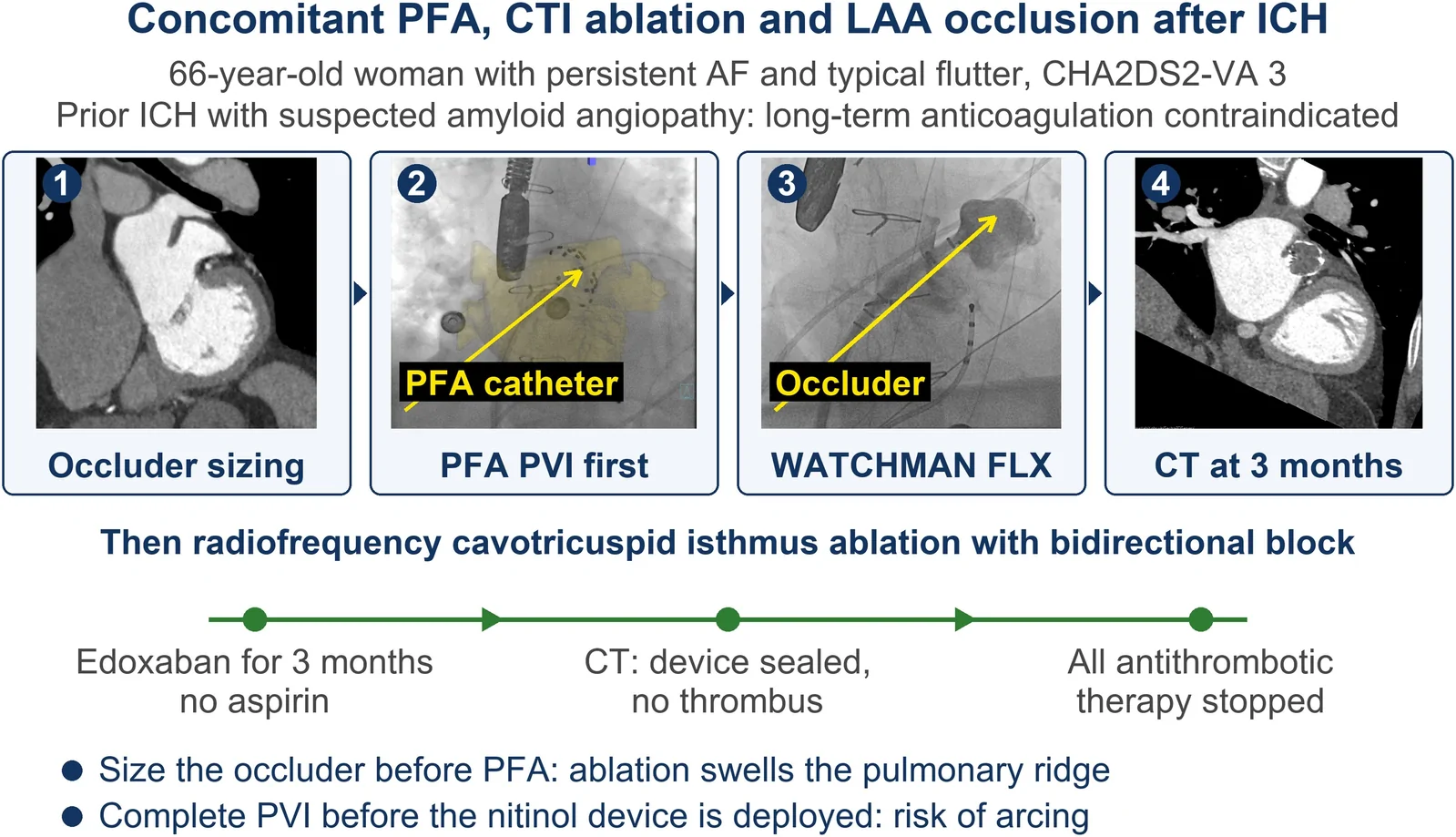
Concomitant pulsed field ablation, cavotricuspid isthmus ablation, and left atrial appendage occlusion after intracranial haemorrhage.

## Case presentation

A 66-year-old woman was referred for management of atrial arrhythmia and stroke prevention. She had a long history of persistent AF with successful cardioversions in 2015 and 2022, and more recently symptomatic, recurrent cavotricuspid isthmus–dependent atrial flutter (*Figure 1*). Brief sensorimotor disturbances had previously been attributed to possible transient ischaemic attacks. Her CHA_2_DS_2_-VA score was 3 (age 65–74 years and prior transient ischaemic attack). She had not received anticoagulation for 2 years following an ICH in 2023, after which brain magnetic resonance imaging (MRI) had shown microhaemorrhages and siderosis suggestive of CAA. Other history included surgical closure of a secundum atrial septal defect at age 30 for right ventricular dilatation and dysfunction.

**Figure 1:**
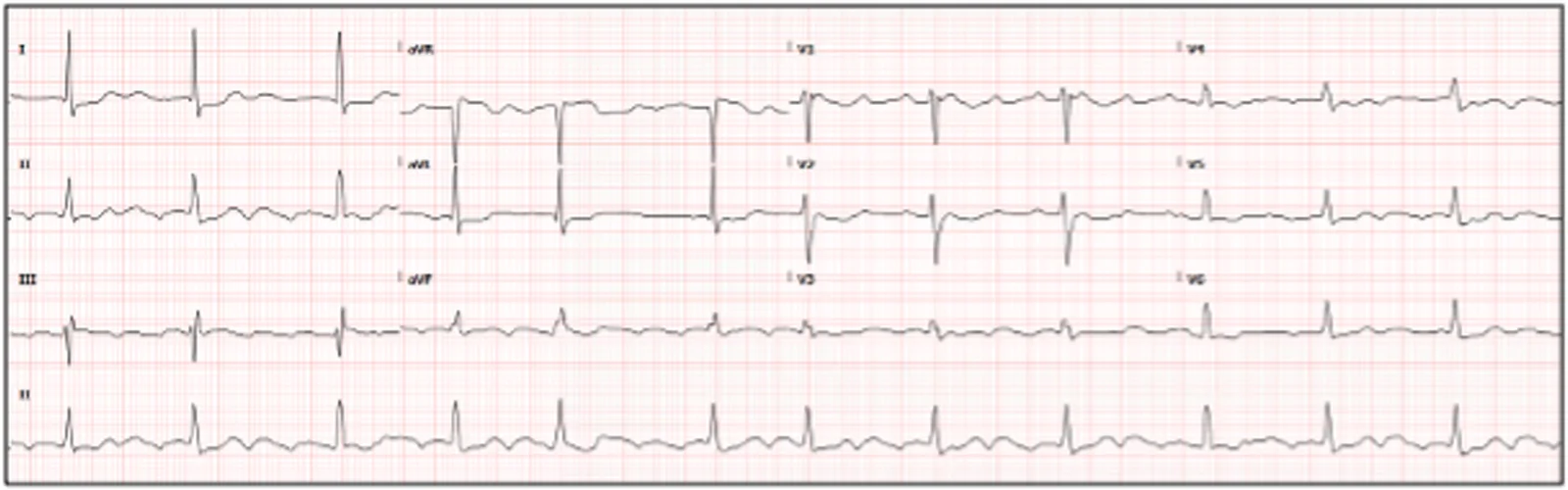
12-Lead electrocardiogram demonstrating atrial flutter (found to be cavotricuspid isthmus dependent/typical right atrial flutter).

Pre-procedural cardiac computed tomography (CT) showed a single-lobed left atrial appendage (LAA) suitable for a WATCHMAN FLX device (Boston Scientific), from which the device was sized before ablation (*Figure 2*). The procedure was performed under general anaesthesia with transoesophageal echocardiographic (TOE) guidance. Two ultrasound-guided right femoral venous punctures were performed. A quadripolar diagnostic catheter was positioned in the coronary sinus for pacing and recording (visible in *Figures 3* and *4*). Transseptal access was obtained through the FARADRIVE sheath (Boston Scientific) with a 98 cm Brockenbrough needle and SafeSept wire,^1^ targeting an inferior puncture site to facilitate later LAA access. Heparin was administered to maintain an activated clotting time above 300 s. To avoid electrical interaction with the nitinol occluder frame, pulmonary vein isolation (PVI) was performed first with the pentaspline PFA catheter (FARAPULSE, Boston Scientific) under CT–fluoroscopy merge guidance (Syngo, Siemens) (*Figure 3*), delivering the standard 32-application lesion set for ostial and antral isolation of all four veins.

**Figure 2:**
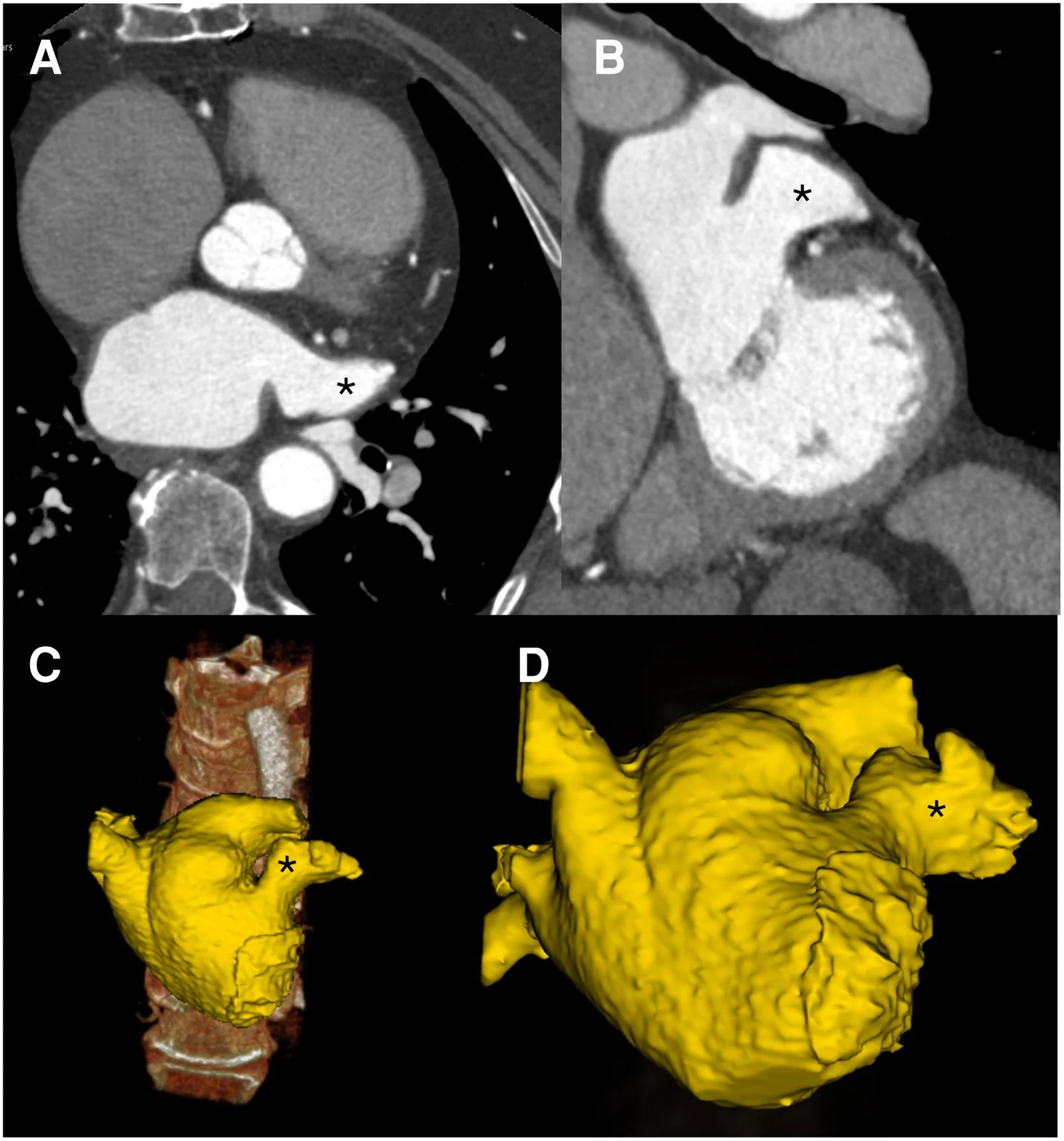
Pre-procedural computed tomography assessment of the left atrial appendage. Panel A shows an axial slice of the left atrial appendage (which opacifies with contrast indicating the absence of thrombus. Panel B shows a coronal plane of the left atrial appendage viewed from the patient’s right. Panels C and D show three-dimensional reconstructions of the left atrium with the left atrial appendage and pulmonary veins demonstrated. The left atrial appendage is indicated by the *.

**Figure 3:**
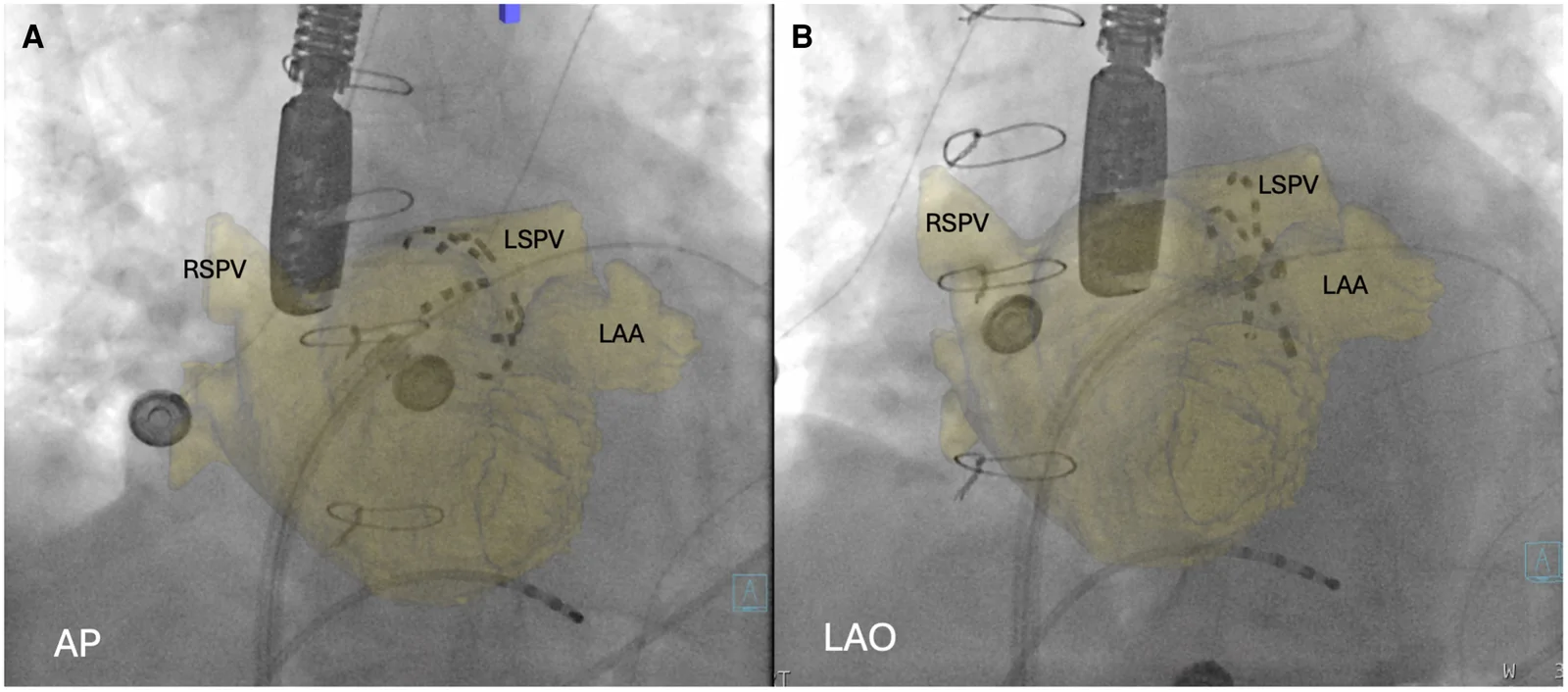
Pulmonary vein isolation using pentaspline pulsed field ablation catheter. Pulsed field ablation with the FARAPULSE system (Boston Scientific) to isolate all four pulmonary veins. The yellow outline represents merging of a computed tomography three-dimensional segmentation with the fluoroscopy system (Syngo, Siemens). The transoesophageal echocardiographic probe and the quadripolar diagnostic catheter in the coronary sinus are also visible. Panel A shows the catheter in ‘basket’ configuration in the left superior pulmonary vein. Panel B shows the catheter in ‘flower’ configuration in the left inferior pulmonary vein. LAA, left atrial appendage; RSPV, right superior pulmonary vein.

**Figure 4:**
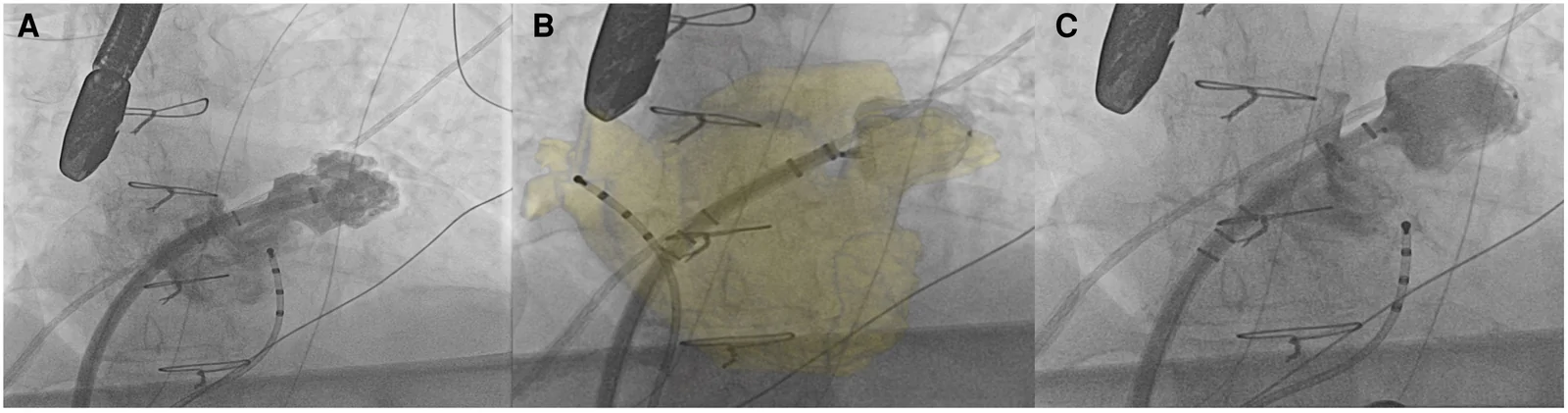
Deployment of the percutaneous left atrial appendage occlusion device. Deployment of a WATCHMAN FLX (Boston Scientific) percutaneous left atrial appendage occlusion device. Panel A shows contrast opacification of the left atrial appendage prior to deployment. Panel B shows deployment of the left atrial appendage occlusion device using a merged computed tomography segmentation. Panel C shows contrast opacification of the device, demonstrating the absence of peri-device leak after deployment. The quadripolar diagnostic catheter and transoesophageal echocardiographic probe are also visible.

The FARADRIVE sheath was then exchanged for the WATCHMAN FLX delivery sheath, which was advanced into the LAA over a 6 F pigtail catheter to avoid cardiac injury. Contrast opacification of the LAA was aligned with the merged CT to guide deployment (*Figure 4*). Position, anchor, size, and seal criteria were met on TOE and fluoroscopy, and the device was released. The delivery sheath was withdrawn to the right atrium, and cavotricuspid isthmus ablation was performed with a 3.5 mm irrigated contact-force radiofrequency catheter (THERMOCOOL SMARTTOUCH, Biosense Webster) advanced through the FARADRIVE sheath. Bidirectional isthmus block was confirmed by differential pacing from the coronary sinus and ablation catheters. There were no procedural or access-site complications. She was discharged the following morning on edoxaban 60 mg once daily for 3 months, without antiplatelet therapy. This course covered the period of highest risk of device-related thrombus (DRT) and the post-ablation window for which at least 2 months of anticoagulation is recommended. Edoxaban was chosen as the locally preferred once-daily agent.

At 3 months, cardiac CT confirmed a well-seated device without peri-device leak or DRT (*Figure 5*). There had been no bleeding or neurological events, and dyspnoea and palpitations had resolved. Edoxaban was stopped, and aspirin was not started, given its additional haemorrhagic risk in CAA and the absence of any other indication for it.

**Figure 5:**
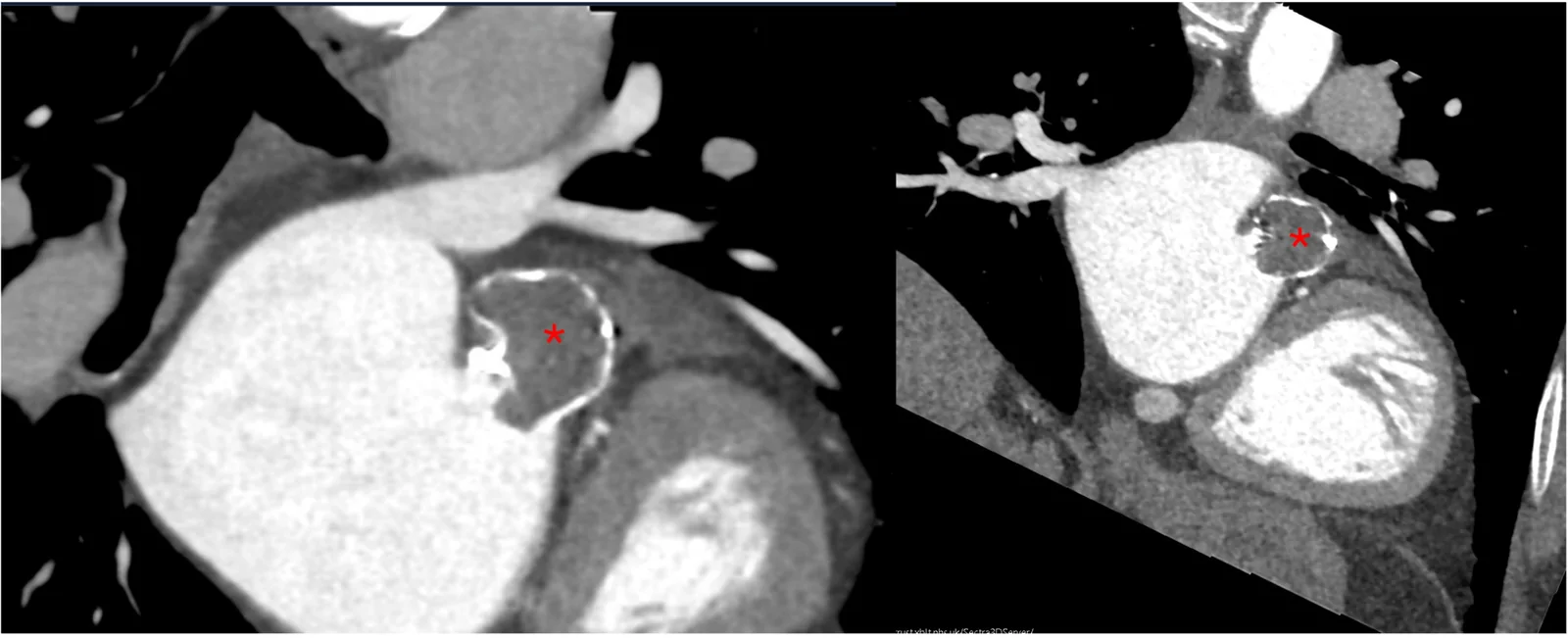
Follow-up cardiac computed tomography at 3 months to assess left atrial appendage occlusion. Two views demonstrating complete occlusion of the distal left atrial appendage (*) without evidence of contrast leak or excessive device protrusion from the left atrial appendage.

## Discussion

Current ESC guidelines recommend long-term OAC for patients with a CHA_2_DS_2_-VA score of two or more, irrespective of rhythm-control interventions such as catheter ablation. However, they also identify intracerebral haemorrhage related to CAA as one of the few absolute contraindications to OAC, and state that pLAAO may be considered in patients with such contraindications.^2^

The distinctive feature of this case is the setting of prior ICH with suspected CAA. CAA-related haemorrhage carries a high risk of recurrence, and the microbleed burden and siderosis on this patient's MRI are recognized predictors of further bleeding.^3^ In PRESTIGE-AF, direct oral anticoagulants almost eliminated ischaemic stroke in ICH survivors with AF (1 vs. 20 events) but increased recurrent ICH more than 10-fold (hazard ratio [HR] 10.89, 95% confidence interval [CI] 1.95–60.72),^4^ and ENRICH-AF stopped enrolling patients with lobar ICH or convexity subarachnoid haemorrhage because of excess recurrent haemorrhage with edoxaban.^5^ Aspirin has also been associated with recurrence after lobar ICH.^6^ Observational series suggest pLAAO is feasible in this population, with pooled ischaemic stroke and recurrent ICH rates of approximately 2% each over a mean follow-up of 17 months, and roughly double that in the subgroup with intraparenchymal haemorrhage or CAA.^7^ Ablation was indicated for symptoms but would not have removed the need for stroke prevention, and combining the procedures provided both through one transseptal access and one time-limited period of anticoagulation rather than two procedures each requiring peri-procedural OAC.

Antithrombotic therapy after pLAAO in this setting has no dedicated evidence base and must balance DRT, which is most frequent before device endothelialization, against recurrent haemorrhage. Rather than the OAC-plus-aspirin regimen followed by long-term aspirin used in OPTION,^8^ we used a single short course of edoxaban without aspirin and withdrew all antithrombotic therapy once CT confirmed seal without DRT, accepting a small risk of late DRT to avoid the haemorrhagic risk of indefinite aspirin. No direct oral anticoagulant has shown superiority over another after ICH, and the ENRICH-AF experience argues that any such anticoagulation should be as brief as possible.^5^ Heeger *et al*. recently described a similar single-access PFA and WATCHMAN FLX procedure in an 82-year-old man with persistent AF and prior gastrointestinal bleeding, treated with apixaban for 2 months and then long-term aspirin.^9^ Our case differs in the indication, in which even antiplatelet therapy carries risk, and in the complete withdrawal of antithrombotic therapy after imaging confirmation of seal.

The OPTION trial supports concomitant pLAAO with AF ablation.^8^ pLAAO was non-inferior to OAC for the composite of all-cause mortality, stroke or systemic embolism and superior for the primary safety endpoint of non-procedural major bleeding.^8^ Outcomes in the concomitant cohort (pLAAO within 10 days of ablation) matched those of sequential procedures without additional peri-procedural morbidity.^8^

OPTION’s endpoint design has nonetheless drawn criticism. Excluding procedural bleeding from the primary safety endpoint favours a device that carries upfront procedural risk, and for all international society on thrombosis and haemostasis (ISTH) major bleeding the device met non-inferiority but not superiority (HR 0.77, 95% CI 0.48–1.24).^8^ CLOSURE-AF, which compared pLAAO with best medical therapy in patients at high bleeding risk, found higher major bleeding with pLAAO when peri-procedural events were included (7.4% vs. 6.2%).^10^ This incremental hazard is smaller in patients already undergoing transseptal ablation.

The efficacy composite is also debated, because all-cause mortality dilutes a stroke signal that was low in both arms (1.2% vs. 1.3%).^8^ OPTION also excluded patients with a bleeding-related contraindication to OAC, so patients such as ours remain unrepresented in randomized data.

Integrating PFA with pLAAO introduces considerations not captured in OPTION, which used predominantly thermal ablation. Tam *et al*. reported high implantation success for combined PFA and pLAAO but a 69.4% mean increase in pulmonary ridge thickness immediately after ablation.^11^ Late peri-device leak (>3 mm) at 3 months was consequently more frequent in the combined cohort (20% vs. 2.2%), reflecting device undersizing.^11^ Heeger *et al*. observed the same ridge swelling after PVI.^9^ The occluder should therefore be sized from pre-ablation imaging, as in this case, rather than from post-ablation measurements.

These findings also bear on procedural sequencing. Deploying the occluder before PVI would avoid post-ablation oedema, but PFA may interact electrically with the nitinol frame.^11^ PFA delivers ultra-short, high-voltage pulses, and applications near a metallic implant risk arcing, thermal injury, loss of device integrity, and unpredictable lesion formation.^12^ PVI should therefore be completed before the occluder is introduced.

This case illustrates a pragmatic single-procedure approach to rhythm control and stroke prevention in a patient in whom prior ICH with suspected CAA made long-term OAC unacceptable. This population is absent from current trials, and ongoing studies will clarify the role of pLAAO after ICH.^13^

## Data Availability

There is no data to be made available for this report.

## Lead author biography

Dr. Rahul Muthalaly is a cardiac electrophysiology fellow based at Barts Heart Centre in the United Kingdom. He trained in Australia at the Victorian Heart Hospital. He has a special interest in cardiac CT, atrial fibrillation, ventricular tachycardia, and statistics.
